# Psoriasis treatments in the stabilization of atherosclerosis: a systematic review

**DOI:** 10.1007/s00403-024-03625-6

**Published:** 2024-12-30

**Authors:** Lixin Ji, Sowmya Ravi, Laura Wright, Vi Nguyen, Jose Wiley, Milena Vukelic, Sangkyu Kim

**Affiliations:** 1 Department of Dermatology, Tulane School of Medicine, 131 S. Robertson Street, 11th Floor, New Orleans, LA 70112 USA; 2 Tulane School of Medicine, Rudolph Matas Library of Health Sciences, New Orleans, LA USA; 3 Department of Surgery, Tulane School of Medicine, New Orleans, LA USA; 4 Department of Cardiology, Tulane School of Medicine, New Orleans, LA USA; 5 Department of Rheumatology, LSU School of Medicine, New Orleans, LA USA; 6 Department of Biomedical Sciences, Tulane School of Medicine, New Orleans, LA USA

**Keywords:** General dermatology, Medical dermatology, Clinical research, Psoriasis, Complex medical dermatology, Atherosclerosis

## Abstract

This systematic review explores the relationship between achieving minimal disease activity in psoriasis and the progression of atherosclerosis. It investigates how biologic therapies and other treatments impact atherosclerosis markers, offering insights into therapeutic strategies. A comprehensive search of PubMed, Embase, and Web of Science was conducted from January 1, 2000, to April 1, 2023, using terms such as psoriasis, psoriatic arthritis, atherosclerosis, biologic therapy, vascular stiffness, carotid intima-media thickness (CIMT), and coronary computed tomography angiography (CCTA). Eligible studies were those involving human subjects over 18, written in English, that provided quantitative atherosclerosis markers, including CIMT, CCTA, arterial pulse wave velocity (aPWV), fat attenuation index (FAI), and augmentation index (Aix). From an initial pool of 217 studies, 21 were included, grouped by treatments, including TNF-α inhibitors, IL-12/23 inhibitors, IL-17 inhibitors, DMARDs, phototherapy, and fumaric acid esters. The review found that TNF-α inhibitors significantly improved atherosclerosis markers such as CIMT, aPWV, FAI, and C-reactive protein (CRP), with reductions in these markers compared to no treatment, phototherapy, and IL-12/23 inhibitors. Additionally, IL-17 inhibitors demonstrated similar reductions in FAI compared to TNF-α inhibitors but showed a greater effect in reducing non-calcified plaque burden (12% vs. 5% for TNF-α inhibitors, p < 0.001) and also decreased CRP levels. Fumaric acid improved cholesterol metabolism (p < 0.04), and TNF-α inhibitors enhanced endothelial function (p < 0.01). Mixed results were observed when compared to DMARDs, indicating that patient-specific factors should guide treatment choices. In conclusion, TNF-α inhibitors are highly effective in reducing atherosclerosis progression in psoriasis patients, consistently improving vascular health markers like CIMT, aPWV, FAI, and CRP. IL-17 inhibitors also show significant efficacy, particularly in reducing non-calcified plaque burden, making them a valuable alternative to TNF-α inhibitors. Fumaric acid’s role in cholesterol metabolism suggests its potential in combination therapies. These findings support integrating TNF-α and IL-17 inhibitors into treatment protocols for psoriasis patients with comorbid atherosclerosis, improving cardiovascular outcomes.

**Supplementary Information:**

The online version contains supplementary material available at 10.1007/s00403-024-03625-6.

## Introduction

Psoriasis is a chronic immune-mediated inflammatory disorder affecting approximately 125 million people globally [1] It typically manifests as skin plaques, but 15–30% of patients also develop severe disease phenotypes, including nail involvement and peripheral and axial arthritis (PsA) [2]. Population-based studies have revealed a threefold increase in the risk of atherosclerosis, myocardial infarction, stroke, and cardiovascular-related deaths among patients with psoriasis and PsA, even after accounting for traditional risk factors commonly associated with psoriasis, such as diabetes, obesity, hypertension, and metabolic syndrome [3–5]. The risk intensifies in patients with severe disease phenotypes, reflecting a high burden of inflammation driven by elevated levels of proinflammatory cytokines, such as TNF-α, IL-17, and IL-23, which contribute to endothelial dysfunction, plaque formation, and atherosclerotic development [6–11] .

Atherosclerosis is recognized as a chronic inflammatory condition characterized by abnormal innate and adaptive responses to various antigens and cytokine production [6, 7]. This understanding was pivotal in the Canakinumab Anti-Inflammatory Thrombosis Outcomes Study (CANTOS) trial, which demonstrated that canakinumab, an antibody that blocks IL-1β, significantly reduced the rate of recurrent cardiovascular events. [12]

Biologic therapies are widely used to manage severe psoriasis of the skin and nails, as well as the musculoskeletal manifestations of PsA [13, 14]. Often, these are complemented by non-steroidal anti-inflammatory drugs, methotrexate, sulfasalazine, and apremilast. Considering the shared pathological mechanisms between psoriasis and atherosclerosis, a critical clinical question arises: Can biologic agents slow the progression of coronary artery plaques in patients? Review of the literature indicates that biologics, such as TNF-α inhibitors, IL-12/IL-23 inhibitors, and anti-IL-17 inhibitors, generally have favorable effects on atherosclerosis in patients with psoriasis and PsA [15–19].

This review will analyze studies grouped by the following treatments and further categorized by study design:

1. TNF-α inhibitors versus no treatment.
2. TNF-α inhibitors versus IL-17 inhibitors.
3. TNF-α inhibitors versus IL-12/23 inhibitors.
4. IL-17 inhibitors vs IL-12/23 inhibitors.
5. TNF-α inhibitors versus DMARDs, such as methotrexate.
6. TNF-α inhibitors versus phototherapy.
7. TNF-α inhibitors versus fumaric acid esters.

This systematic review will also assess the most common methods used to measure atherosclerosis, including carotid intima-media thickness (CIMT), coronary computed tomography angiography (CCTA), arterial pulse wave velocity (aPWV) for assessing arterial stiffness, and augmentation index (Aix). Specifically, the review will delve in to how these parameters change with the different treatments that target psoriasis and PsA.

## Methods

### Data sources and search strategy

This review is compliant with PRISMA 2020 guidelines. We utilized 3 databases to ensure comprehensive results: PubMed, Embase (Elsevier), and Web of Science (Clarivate). One author (LJ) wrote the protocol. One author (LJ) in consultation with a health sciences librarian (LAW) established search terms for each database. The search was conducted in April 2023. The original search strategy was created in PubMed and translated to the other databases. Search terms included psoriasis, psoriatic arthritis, atherosclerosis, biological therapy, biological factors, therapeutics, vascular stiffness, carotid intima-media thickness, and coronary computed tomography angiography. Where appropriate, database-specific search terms were used (e.g. MeSH, Emtree). A complete list of search strategies is provided in eTable 1, which is available in the supplemental file. We searched the databases from Jan 1, 2000, to April 1, 2023. During the search, 217 studies were collected in total initially, 156 irrelevant after title and abstract screening, which left the team with 61 papers. After full text review, 17 were excluded, which left the team with 44 studies. Out of those studies, 23 had no control groups, which left the team with a final 21 papers for our systematic review.

## Results

Our review revealed the following. Nine papers compare patients given TNF-α inhibitors vs no treatments [15, 19–26]. Four papers study patients given TNF-α inhibitors vs DMARDs [27–30]. One paper compares patients given TNF-α inhibitors vs anti IL-12/23 [31]. Two papers study patients given TNF-α inhibitors vs phototherapy [16, 32].

The most common way to access atherosclerosis is carotid intima-media thickening (CIMT) using high-definition ultrasound and arterial tonometry. CIMT is a measure of the thickness of the inner two layers of the carotid artery and reflects the progression of atherosclerosis [25]. The other common parameters are coronary computed tomography angiography (CCTA), aortic pulse wave velocity (aPWV), aortic augmentation index (Alx), coronary artery calcium (CAC) computed tomography (CT) and flow-mediated dilation (FMD). CCTA is an imaging that detects plaque buildup [17]. aPWV measures the speed at which the blood pressure pulse travels through the arteries and is a standard method for determining arterial stiffness [25]. Alx indicates arterial stiffness by measuring the pressure added by the reflected blood wave returning to the heart [25]. CAC quantifies the amount of calcium in the coronary arteries [17]. FMD assesses endothelial function by measuring how blood vessels widen in response to increased blood flow. [33]

Moreover, inflammatory markers, such as C-reactive protein (Crp) and erythrocyte sedimentation rate (ESR) have been used by six papers to assess inflammation levels in patients [16, 19, 23–25, 33].

### TNF-α inhibitors vs no treatments

*Randomized Clinical Trials (RCTs):* Five RCTs have been conducted, all reporting that TNF-α inhibitors positively impact the progression of atherosclerosis in patients with psoriasis, PsA, and other rheumatoid diseases [15, 23–26]. The parameters used in these studies include arterial pulse wave velocity (aPWV), carotid intima-media thickness (CIMT), coronary computed tomography angiography (CCTA), augmentation index (Alx), and C-reactive protein (CRP) [15, 23–26]. Significant reductions in aPWV, CIMT, and CRP were observed in the treatment groups, contrasting with increases in the non-treatment groups [15, 24–26]. Alx, however, did not change in any of the groups [23]. Additionally, one study noted that CCTA was stable in the treatment group and worsened in the non-treatment group [15].

*Prospective Cohort Studies*: There are three such studies, all indicating that TNF-α inhibitors were associated with improved atherosclerosis metrics [19, 21, 22]. Two of these studies specifically measured total plaque area, revealing significant reductions in non-calcified plaque burden and necrotic core [19, 22]. Another study used CCTA to assess coronary plaque and reported a reduction in overall non-calcified plaque burden [21].

*Controlled Study*: The last study in this category focused on patients given TNF-α inhibitors versus no treatments, showing that the treatment group maintained stable CAC scores while the control group showed increases in CAC scores and enhanced plaque formation in coronary arteries [20].

### TNF-α inhibitors vs IL-17 inhibitors

There are two perspective cohort studies that have compared the effects of TNF-α inhibitors with IL-17 inhibitors, a relatively newer biologic treatment for psoriasis. In one study, both TNF-α inhibitors and IL-17 inhibitors were shown to reduce the fat attenuation index (FAI), a marker that quantifies coronary inflammation [16]. The reduction in FAI was slightly greater with IL-17 inhibitors compared to TNF-α inhibitors (TNF-α inhibitors: −75.49 [IQR, − 79.12 to − 68.58] at 1 year, P < 0.001; IL-17 inhibitors: − 76.92 [IQR, − 81.16 to − 71.67] at 1 year, P < 0.001) [16]. However, the difference was not statistically significant (P > 0.05), leading the authors to conclude that both TNF-α and IL-17 inhibitors similarly moderate the progression of atherosclerosis in psoriasis patients [16].

The second study, on the other hand, found that IL-17 inhibitors had a more pronounced effect in reducing non-calcified plaque burden compared to TNF-α inhibitors. After one year of therapy, a 5% reduction was observed with TNF-α inhibitors (P = 0.06) and a 12% reduction with IL-17 inhibitors (P < 0.001), suggesting that IL-17 inhibitors may offer superior efficacy in reducing non-calcified plaque burden [19].

### TNF-α inhibitors vs IL-12/23 inhibitors

There is one perspective cohort study compared the effects of TNF-α inhibitors against IL-12/23 inhibitors, revealing that arterial stiffness (AS) and carotid stiffness (CS) were reduced in patients taking TNF-α inhibitors while they increased in those taking IL-12/23 inhibitors (+ 1.15 m/s, p < 0.05) [31]. Carotid distension (CD) decreased in the IL-12/23 group but increased in the TNF-α group (−3.82 kp^−1^10^–3^, p < 0.04) [31]. However, carotid intima-media thickness (CIMT) increased in both groups, more notably in the IL-12/23 inhibitor group, indicating that TNF-α inhibitors may be more effective in combating arterial remodeling [31].

### IL-17 inhibitors vs IL-12/23 inhibitors

There is one prospective cohort study that demonstrated that IL-17 inhibitors had a greater impact on reducing non-calcified plaque burden compared to IL-12/23 inhibitors, with a 12% reduction in the IL-17 group (P < 0.001) versus a 2% reduction in the IL-12/23 group (P = 0.36) after one year of therapy [19]. The study concluded that IL-17 inhibitors not only produce a more significant reduction in non-calcified plaque burden than TNF-α inhibitors, but also outperform IL-23 inhibitors in this regard. [19]

### TNF-α inhibitors vs DMARDs

There are four observational studies that examine the atherosclerosis progression in patients treated with TNF-α inhibitors versus methotrexate [27–30]. Two studies found that TNF-α inhibitors led to more stable atherosclerosis metrics compared to methotrexate [27, 30]. Specifically, these patients had lower CIMT and improved flow-mediated dilation (FMD) [27, 30]. Another study highlighted that while both biologic and systemic therapies positively affected arterial remodeling, methotrexate showed a more significant reduction in CIMT and improvements in glucose and insulin levels [29]. Contrarily, a fourth paper reported that the positive effects of TNF-α inhibitors on FMD were transient, diminishing over 2–3 months, presenting mixed outcomes across these studies [28].

### TNF-α inhibitors vs phototherapy

There are two pilot and prospective cohort studies that assessed the comparative effectiveness of TNF-α inhibitors and phototherapy on atherosclerosis [16, 32]. The findings indicated that TNF-α inhibitors more effectively reduced coronary inflammation, evidenced by significant reductions in CIMT, coronary plaques (measured by CCTA), CRP, and Psoriasis Area and Severity Index (PASI) scores compared to phototherapy [16, 32].

### TNF-α inhibitors vs fumaric acid

*There is one randomized clinical trial* explores the differential impacts of TNF-α inhibitors and fumaric acid on atherosclerosis [33]. Results showed that TNF-α inhibitors significantly enhanced FMD and reduced high-sensitivity C-reactive protein (hsCRP), whereas fumaric acid significantly lowered total cholesterol and apolipoprotein B levels, suggesting a beneficial effect on cholesterol metabolism. The contrasting outcomes demonstrated that both treatments offer distinct cardiovascular benefits in psoriasis patients, with adalimumab primarily improving inflammatory markers and endothelial function, and fumaric acid esters (FAE) enhancing cholesterol metabolism [33].

It Is Important to look at how different metrics change in different treatment groups to gauge the impact of treatments on the progression of atherosclerosis. This section delves into the modifications in key cardiovascular metrics—Carotid Intima-Media Thickness (CIMT) and Arterial Pulse Wave Velocity (aPWV) and fat attenuation index (FAI)—in response to various biologic treatments. Additionally, given the inflammatory nature of both psoriasis and atherosclerosis, this discussion extends to evaluating changes in inflammatory markers, namely augmentation Index (Aix), c-reactive protein (CRP) and erythrocyte sedimentation rate (ESR), to understand the broader implications of biologic therapies on systemic inflammation and vascular health.

### Carotid intima-media thickness (CIMT)

Our comprehensive review includes nine studies that explore the progression of CIMT under various treatment modalities [25, 28, 30–34]. A pivotal study assessing the effects of TNF-alpha inhibitors versus no treatment documents a substantial reduction in CIMT progression after 12 months of therapy and an increase in CIMT in the control group (baseline: 0.551 [0.462, 0.660] mm, 12 months: 0.569 [0.499, 0.668] mm, *P* = 0.02), highlighting the treatment’s efficacy (Table 1) (Table 1) [25]. In contrast, a study comparing TNF-alpha inhibitors with anti-IL-12/23 shows an increase in CIMT in both groups, more significantly in the anti-IL-12/23 group (Table 1) [31]. This suggests an adverse trend associated with these therapies. When analyzed against phototherapy that exhibits a 13.7% increase in right CIMT, TNF-alpha inhibitors demonstrate a 4.3% reduction in right CIMT, suggesting superior efficacy in managing arterial health [from 0.630 mm to 0.588 mm (left CIMT)] (Table 1) [32].

**Table 1:** Studies evaluating the effect of TNF-α antagonists on intima-media thickness in patients with psoriasis and inflammatory arthritis

Author	Year	Disease	Patients (n)	Age (years)	Disease duration (years)	Drug used	Follow-up duration (months)	Study design	Change in CIMT	Effect
Angel et al	2012	RA, AS, PsA	55	TNF-α inhibitors: 47.2 ± 11.6; control: 51.9 ± (14.5)	11	TNF-α inhibitors vs no treatment	12	RCT	−0.002 mm (0.038, 0.030) in TNF-treated group; 0.030 mm (0.011, 0.043) in control	↓ in TNF-treated group, ↑ in control
Aleissa et al	2020	PV	29	50	20.9	TNF-α inhibitors vs UST	18	OBS	Increased in both groups, more importantly in the UST group	↑ in both groups
Blasco-Morente et al	2018	Psoriasis	14	45.3 ± 6	17.2 ± 4.5	TNF-α inhibitors vs Phototherapy	12	OBS	Right CIMT: 0.610 mm to 0.604 mm, P = 0.037; Left CIMT: 0.630 mm to 0.588 mm, P = 0.046	↓ in both right and left CIMT in TNF-treated group
Di Minno et al	2011	PsA	224	52.61 ± 11.37	109.04 ± 71.9	TNF-α inhibitors vs DMARDs (methotrexate, sulfasalazine, cyclosporine A, leflunomide)	12	OBS	Carotid plaques were detected in 15.8% of those on TNF-α blockers and in 40.4% of those on DMARDs	Significant ↓ in TNF-treated group
Mazzoccoli et al	2010	PsA	36	52 ± 9.8	7.2	TNF-α inhibitors vs DMARDs (methotrexate, leflunomide, and sulfasalazine)	3	RCT	N/A	no statistically significant difference among the groups
Ortolan et al	2020	PsA	32	51 ± 8	12 ± 10	TNF-α inhibitors vs DMARDs	24	OBS	CIMT- mean and M-MAX (0.7 ± 0.1 vs. 0.9 ± 0.4 and 0.9 ± 0.2 vs. 1.1 ± 0.4, p < 0.01) after one year of treatment	Significant ↑ at 1 year, stable at 2 years
Lopez	2018	Psoriasis, PsA	53	47.92	17.33	TNF-α inhibitors vs IL-23 inhibitors vs DMARDs	8	OBS	TNF-α inhibitors: no significant decrease in CIMT; a significant decrease in CIMT with IL-23 inhibitors (from 658.18 to 583.59, p = 0.005) and DMARDs (from 570.33 to 523.65, p = 0.045)	Significant ↓ with IL-23 inhibitors DMARDs
Holzer et al	2018	RA, PsA, AS	65	TNF-α inhibitors: 46.3 (11.2); FAE: 43.5 (12.0)	TNF-α inhibitors: 11.9 (11.3); FAE: 10.1 (8.8)	TNF-α inhibitors vs Fumaric Acid Esters (FAE)	6	OBS	TNF-α inhibitors: from 0.559 (0.131) to 0.571 (0.129), P = 0.342; FAE: from 0.530 (0.148) to 0.533 (0.118), P = 0.21	No significant decrease with TNF-α inhibitors or FAE
Tam et al	2011	PsA	20	N/A	N/A	TNF-α inhibitors	3	RCT	Group 1: from 0.75 to 0.81 (0.70–0.90) mm (0.65–0.85 mm, P = 0.003; Group 2: from 0.78(0.70–0.82) mm to 0.78 (0.71–0.84) mm, P = 0.085; Group 3: from 0.87 (0.82–1.12) mm to 0.90 (0.81–1.15) mm, P = 0.239)	↓ in Group 1 (TNF-α inhibitors over 12 weeks), « in Group 2 (TNF-α inhibitors less than 12 weeks), ↑ in group 3 (no treatment)

Further analysis in one study reveals that among PsA patients, those on TNF-alpha blockers exhibit lower CIMT and fewer carotid plaques compared to those on traditional DMARDs, highlighting its potential in preventing atherosclerotic changes (Table 1) [30]. However, contrasting TNF-alpha inhibitors with DMARDs in three other studies shows the contrary. The first study shows that there is no significant change in CIMT values between two groups. The second study shows a similar finding with an additional finding that patients have a more significant decrease in CIMT IL-23 inhibitors and DMARDs. The third study exhibits that there is a significant increase in both IMT-mean and IMT-max during the first year of anti-TNF-α treatment, and they become stable after two years of treatment [27, 28]. Comparisons with fumaric acid esters reveal no significant differences in CIMT, indicating comparable efficacy [33]. Lastly, duration-dependent effects analyzed in one study reveal that sustained improvement in CIMT requires continuous TNF-alpha blocker therapy beyond 12 weeks [34].

### Arterial pulse wave velocity (aPWV)

The progression of aPWV is assessed in four studies, all of which demonstrate that TNF-alpha inhibitors significantly reduce aPWV in the treatment group, with no changes observed in the control group [23–26]. This consistent reduction highlights the potential of TNF-alpha inhibitors in ameliorating arterial stiffness, a critical factor in cardiovascular health (Table 2).

**Table 2:** Studies evaluating the effect of TNF-α Antagonists on aPWV in patients with psoriasis and inflammatory arthritis

Author	Year of publication	Disease	Patients, n	Age, years	Disease duration, years	Drug used	Follow-up duration, months	Study design	Effect	Change in aPWV
Angel et al	2010	RA, AS, PsA	60	47.0 ± 12.3 years (anti-TNF group)/51.7 ± 14.6 years (control group)	10.5 ± 7.9 (TNF- α inhibitors)/11.2 ± 8.4 (control group)	TNF-α inhibitors vs no treatment	12	RCT	↓	− 0.50 ± 0.78 m/s for treatment group/0.05 ± 0.54 m/s for control; P = 0.002
Angel et al	2012	RA, AS, PsA	55	TNF-α inhibitors: 47.2 ± 11.6; control: 51.9 ± (14.5)	10.1 ± 8.8 TNF- α inhibitors/11.9 ± 11.3 (control group)	TNF-α inhibitors vs no treatment	12	RCT	↓	− 0.54 (0.79)/0.06 (0.61) m/s P = 0.004
Angel et al	2009	RA, AS, PsA	36	46.2 (12.2)/49.0 (14.1)	11.0 (9.6)/11.6 (10.1)	TNF-α inhibitors vs no treatment	12	CCS	↓	− 0.53/0.08 m/s, P = 0.001
Angel et al	2010	RA, AS, PsA	60	N/A	N/A	TNF-α inhibitors vs no treatment	12	RCT	↓	− 0.52 ± 0.80/0.04 ± 0.48 m/s, P = 0.001

### Fat attenuation index (FAI)

One paper examines how Fat Attenuation Index (FAI) changes with biologic therapy and shows a significant decrease in FAI in patients after one year of treatment with biologic agents, including TNF-α inhibitors, anti–IL-12/23 inhibitors, and anti–IL-17 inhibitors. There is no significant change in FAI observed in the control group. Furthermore, the paper demonstrates that the decrease in FAI is consistent across the three biologic therapies—TNF-α inhibitors, anti–IL-12/23 inhibitors, and anti–IL-17 inhibitors. In other words, these biologic agents have similar effects on FAI.

### Augmentation index (Aix)

There are three papers look at how Aix changes with biologic therapy, and they all compare patients who are given TNF-α inhibitors vs no treatment. These results consistently indicate that while TNF-α inhibitors can improve measures of arterial stiffness like CIMT and aPWV, it does not significantly impact Aix [23–25].

### C-reactive protein (CRP)

Our analysis of six studies exploring changes in CRP levels elucidates the anti-inflammatory effects of TNF-alpha inhibitors [16, 19, 23–25, 33]. Four studies comparing TNF-alpha treatment against no treatment consistently report reductions in CRP levels solely in the treatment groups, reinforcing the anti-inflammatory properties of TNF-alpha inhibitors [19, 23–25]. Another study comparing TNF-alpha with phototherapy observed CRP level reductions only in the TNF-alpha group, further validating the superior anti-inflammatory impact of TNF-alpha inhibitors [16]. Additionally, a comparison between TNF-alpha inhibitors and fumaric acid esters (FAE) notes significant decreases in high-sensitivity CRP (hsCRP) in the treatment group, emphasizing the effectiveness of TNF-alpha inhibitors in reducing systemic inflammation [33].

Another study reports that after one-year of therapy, an improvement in CRP was observed in the anti-IL12/23 and anti-IL17 treated groups (P = 0.02 and P = 0.01; respectively) (Table 3) [19].

**Table 3:** Studies evaluating the effect of TNF-α antagonists on CRP in patients with psoriasis and inflammatory arthritis

Author	Year of publication	Patients (n)	Age (years), anti-TNF/control	Disease duration (years)	Follow-up duration (months)	Drug used	Study design	Effect	Change in CRP (mg/L), treatment/control
Angel et al	2010	60	47.0 ± 12.3/51.7 ± 14.6	10.5 ± 7.9 (Anti-TNF group)/11.2 ± 8.4 (control group)	12	TNF-α inhibitors vs no treatment	RCT	↓	−2.2 (−8.3, 0.0) for treatment/1.5 (1.5,6.0) for control P = 0.001
Angel et al	2012	55	TNF-α inhibitors: 47.2 ± 11.6; control: 51.9 ± (14.5)	10.1 ± 8.8 TNF- α inhibitors/11.9 ± 11.3 (control group)	12	TNF-α inhibitors vs no treatment	RCT	↓	− 5.3 (− 8.1, − 0.1)/ 1.0 (− 4.0, 4.0) P = 0.001
Angel et al	2009	36	46.2 (12.2)/49.0 (14.1)	11.0 (9.6)/11.6 (10.1)	12	TNF-α inhibitors vs no treatment	CCS	↓	−8.63/0.88, P = 0.001
Elnabawi et al. (Coronary)	2019	200	49.1 ± 12.2/51.2 ± 12.0	23.0 ± 14.4/20.3 ± 14.6	12	Biologic treatments (TNF-α inhibitors IL-12/23 inhibitor) vs topical and light therapies	CCS	↓	From 2.0 (0.8–5.0) to 1.4 (0.7–3.6) for treatment group, P < 0.001; from 2.3 (0.6–4.5) to 1.8 (0.7–3.8) for control, P = 0.21
Elnabawi et al	2019	134	49.7 (12.3)/51.4 (12.4)	22.8 (14.1)/ 21.0 (14.1)	12	Biologic treatments (TNF-α inhibitors IL-12/23 inhibitor) vs topical and light therapies	CCS	↓	2.2 (0.8 to 5.5) to 1.3 (0.7 to 3.7), P = 0.03/ from 2.0 (0.6 to 3.5) to 1.5 (0.6 to 3.2), P = 0.12
Holzer et al	2019	65	46.3 (11.2)/ 43.5 (12.0)	11.9 (11.3)/10.1 (8.8)	12	TNF-α inhibitors vs fumaric acid esters	OBS	↓	0.41 (0.40) to 0.34 (0.47), P = 0.022/0.39 (0.38) to 0.39 (0.48), P = 0.595

### Erythrocyte sedimentation rate (ESR)

A singular study examining changes in ESR between patients treated with TNF-alpha inhibitors versus those without treatment found a significant reduction in ESR only in the treatment group. [24] The median changes reported were from 16.0 mm/h (10.5–31.0) to −4.0 mm/h (−12.0– −1.0); P < 0.001, underscoring the treatment's capacity to decrease systemic inflammatory responses, thus contributing to improved patient outcomes in psoriatic and atherosclerotic conditions [24].

## Discussion and limitations

The findings of this comprehensive review underscore the clinical significance of TNF-α inhibitors in managing atherosclerosis, particularly in patients with psoriasis and other rheumatic diseases. The consistent reduction in carotid intima-media thickness (CIMT), aortic pulse wave velocity (aPWV), fat attenuation index (FAI), and inflammatory markers such as C-reactive protein (CRP) highlights the potential of TNF-α inhibitors to not only mitigate the progression of atherosclerosis but also to improve overall vascular health. However, recent studies suggest that other biologic agents, such as IL-17 inhibitors, also play a significant role in managing the cardiovascular risks associated with psoriasis. While TNF-α and IL-17 inhibitors both demonstrate a reduction in FAI, a marker of coronary inflammation, the reduction observed with IL-17 inhibitors was slightly more pronounced, although not statistically significant. Additionally, IL-17 inhibitors have shown superior efficacy over TNF-α inhibitors and IL-12/23 inhibitors in reducing non-calcified plaque burden, suggesting that IL-17 inhibitors may offer added benefits in managing atherosclerosis in this patient population.

The mixed results when compared to DMARDs suggest that while TNF-α inhibitors offer significant benefits, individual patient profiles and comorbidities must guide therapeutic choices. The comparative efficacy of TNF-α inhibitors over IL-12/23 inhibitors and phototherapy further emphasizes the central role of TNF-α inhibitors in comprehensive cardiovascular risk management. Moreover, studies on IL-17 inhibitors present emerging evidence supporting their efficacy in reducing atherosclerotic burden, which highlights the need for continued research and comparison with established therapies. These insights advocate for the integration of both TNF-α inhibitors and IL-17 inhibitors in clinical protocols for patients with concurrent inflammatory and atherosclerotic conditions, promoting a multifaceted approach to treatment that addresses both systemic inflammation and cardiovascular health.

**Fig. 1:**
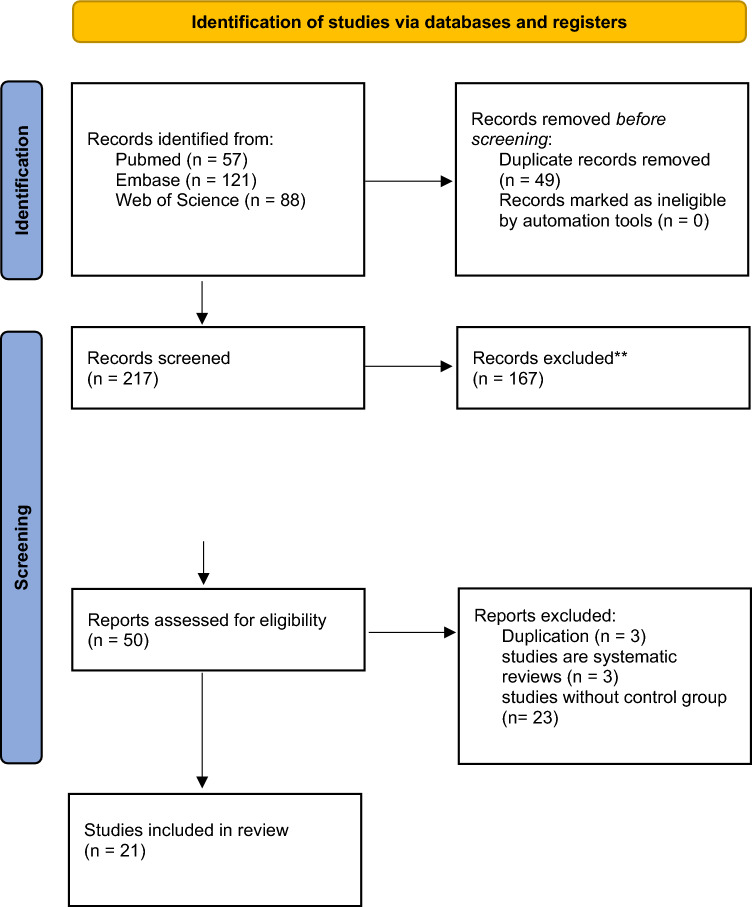
PRISMA Flow Diagramhis is a visual representation of the process used in our systematic review to identify, screen, and include studies. The diagram outlines the stages of the review process, including the number of records identified through database searching and other sources, the number of records after duplicates were removed, the number of records screened, the number of full-text articles assessed for eligibility, and the number of studies included in the final qualitative and quantitative synthesis

This study has several limitations that should be addressed in future research. Firstly, the heterogeneity of the included studies, such as varying study designs, sample sizes, and patient populations, may affect the generalizability of the findings. Additionally, the reliance on different metrics to measure atherosclerosis, like CIMT, aPWV, CRP, and ESR can introduce variability in the results. The duration of follow-up in many studies was relatively short, potentially overlooking long-term effects of TNF-α inhibitors. Moreover, the potential confounding effects of concomitant medications and lifestyle factors were not consistently accounted for across studies. Future research should aim for larger, multicenter randomized controlled trials with standardized atherosclerosis and inflammatory markers. Long-term follow-up studies are necessary to understand the sustained impact of TNF-α inhibitors. Additionally, investigating the effects of combination therapies and the role of patient-specific factors will help tailor treatment strategies for better cardiovascular outcomes (Fig. 1).

## Supplementary Information

Below is the link to the electronic supplementary material.

Supplementary file1 (XLSX 24 KB)

## Data Availability

No datasets were generated or analysed during the current study.
